# Role of cyclooxygenases 1 and 2 in the maintenance of colonic mucosal integrity in an experimental colitis model

**DOI:** 10.1590/1414-431X2023e12946

**Published:** 2023-10-27

**Authors:** H.B. Costa-Filho, T.M.A.L. Sales, S.M. Paula, L.A.D. Nicolau, M.L. Queiroga, A. Havt, P.M.G. Soares, A.L.R. Barbosa, M.H.L.P. Souza

**Affiliations:** 1 Departamento de Fisiologia e Farmacologia, Faculdade de Medicina, Universidade Federal do Ceará, Fortaleza, CE, Brasil; 2 Departamento de Medicina, Faculdade de Medicina, Universidade Federal do Ceará, Fortaleza, CE, Brasil; 3 Centro de Pesquisa em Biotecnologia e Biodiversidade, BIOTEC, Universidade Federal do Delta do Parnaíba, Parnaíba, PI, Brasil; 4 Departamento de Morfologia, Universidade Federal do Ceará, Fortaleza, CE, Brasil; 5 Departamento de Fisioterapia, Universidade Federal do Delta do Parnaíba, Parnaíba, PI, Brasil

**Keywords:** Mucosal integrity, Cyclooxygenase, Colitis

## Abstract

The role of cyclooxygenase (COXs) isoforms in maintaining colonic mucosal integrity is not fully understood. This study aimed to evaluate the role of COX-1 and -2 on colonic mucosal integrity in an experimental colitis model. Colitis was induced in Wistar rats by intracolonic administration of 2,4,6-trinitrobenzenesulfonic acid (20 mg + 50% ethanol). The control group (sham group) received saline only. After 7, 14, or 28 days, colonic samples were removed, and macroscopic lesion scores, wet weight, myeloperoxidase activity, and transepithelial electrical resistance (TER) were determined. In other rat groups, colonic samples from the sham group and a 7th day post-colitis group were mounted in Üssing chambers with the luminal side exposed to a buffer solution (control), acetylsalicylic acid (ASA), SC-560 (COX-1 inhibitor), or celecoxib (COX-2 inhibitor). TER and epithelial permeability to fluorescein were measured. The 7th day colitis group had higher macroscopic damage scores, wet weight, and myeloperoxidase activity and lower basal TER than the sham, 14th day colitis, and 28th day colitis groups. Inhibition of COX-1 but not COX-2 significantly decreased TER and increased permeability to fluorescein in the 7th day post-colitis group compared to the sham group. Additionally, ASA decreased the colonic mucosal integrity on day seven post-colitis compared to the sham group. A decrease in the colonic mucosa integrity in the experimental colitis model can be aggravated only by the inhibition of COX-1, which demonstrated the importance of this enzyme in the maintenance of colonic mucosal integrity.

## Introduction

Inflammatory bowel disease (IBD) is characterized by chronic inflammation of the intestinal mucosa, and includes Crohn's disease (CD) and ulcerative colitis (UC). Studies show that the prevalence of IBD remains high in the Western world, and incidence is increasing in newly industrialized countries (1). In addition to manifestations in the intestinal tract, CD can affect other organs with manifestations outside the intestine, the most common being ophthalmological, dermatological, and rheumatological symptoms (2).

The pathophysiological characteristics of IBD include the presence of pro-inflammatory cytokines, including tumor necrosis factor alpha (TNF-α), interleukin (IL)-1b, IL-6, and IL-8 (3). Additionally, IBD involves alterations in the intestinal mucosal barrier, such as reduction of secretory cells, deficiency of tight junctions (TJs), increased permeability and epithelial reduction, and total loss of epithelia in ulcerated areas. The method used by our research group to induce experimental intestinal inflammation in rodents [2,4,6-trinitrobenzenesulfonic acid (TNBS)-induced colitis] has several aspects that are very similar to the clinical aspects of CD in humans (4,5).

COX-1 is a constitutively expressed enzyme (6) because it is involved in a variety of general physiological activities, including the cellular integrity of the gastrointestinal tract (7). COX-2, in turn, is an enzyme induced in response to cellular activation mediated by hormones, pro-inflammatory cytokines, and growth factors, considered important mediators of the inflammatory process and carcinogenesis (8). Furthermore, studies demonstrate that the levels of COX-2 are elevated during inflammation, being detrimental to the integrity of the intestinal barrier (9). The study of the action of COXs is crucial for understanding the clinical aspects involved in the functional mechanisms of the mucosal barrier. Additionally, the pharmacological approach commonly used against chronic inflammation of the colon, such as non-steroidal anti-inflammatory drugs (NSAIDs), is associated with loss of colonic epithelial integrity, with a decrease in claudin-1, claudin-5, and tricellulin levels and components of TJs. However, to the best of our knowledge, there are no studies on the effect of inhibition of these enzymes on mucosal integrity in IBD (10-12).

Considering that the TNBS colitis model induces severe inflammation and that COXs products are essential to maintaining mucosal integrity, the aim of the present study was to evaluate the effect of specific inhibitors of COX-1 and COX- 2 on the integrity of the colonic mucosa in an experimental model of colitis.

## Material and Methods

### Chemicals

TNBS, non-selective COX inhibitor, acetylsalicylic acid (ASA), selective COX-1 inhibitor (SC-560), ethanol (etOH), fluorescein, hexadecyltrimethylammonium bromide (HTAB), hydrogen peroxide (H_2_O_2_), and O-dianisidine were purchased from Sigma^®^ (Brazil). The selective COX-2 inhibitor (celecoxib) was purchased from Pfizer (Brazil). The salts used for the Krebs solution (sodium chloride, potassium chloride, calcium chloride, magnesium sulfate, monobasic potassium phosphate, sodium bicarbonate, and glucose) were obtained from Dinâmica^®^ (Brazil).

### Animals

Male Wistar rats (190±10 g; aged 6-7 weeks) were obtained from the Animal Facility of the Department of Physiology and Pharmacology of the Federal University of Ceará. The animals were kept in cages under controlled conditions of light (12 h light/dark cycle) and temperature (22±2°C) with free access to water and food. The total number of animals used was 211 and mortality rate was 15%. All treatments and surgical procedures performed were in accordance with the “Guide for the Care and Use of Laboratory Animals” and standards published by the National Council for the Control of Animal Experimentation (CONCEA) and were approved by the Ethics Committee for the Use of Animals of the Federal University of Ceará (CEUA-UFC) (protocol number 128/2017).

### TNBS-induced colitis

**Figure 1:**
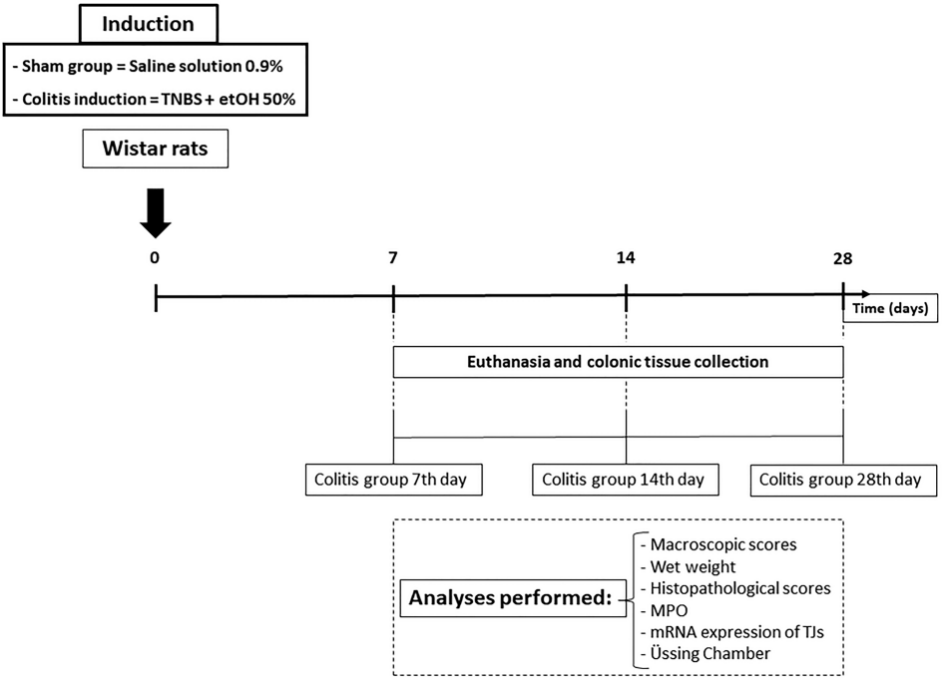
Experimental design. Steps of colitis induction, identification of groups, and collection and processing of colonic tissues. TNBS: 2,4,6,-trinitrobenzenesulfonic acid; MPO: myeloperoxidase.

Initially, the colon of the rats was prepared by removing the feces through an enema (10 mL of 0.9% saline solution, intracolonic). The animals were anesthetized with ketamine (80 mg/kg) and xylazine (10 mg/kg) intraperitoneally. They were placed in the left lateral decubitus position, and the colitis induction solution was administered [0.8 mL TNBS (20 mg) diluted in 50% ethanol, intracolonic] (13). The control animals (Sham) received only saline solution (0.8 mL of 0.9% solution, intracolonic). All intracolonic solutions were administered through a No. 6 polyethylene catheter inserted 8 cm from the anal margin of the rats, and each animal was suspended by the tail for 2 min to prevent the introduced content from flowing back (14). After 7 (7th day colitis group), 14 (14th day colitis group), or 28 (28th day colitis group) days of colitis induction, the animals were euthanized, and colonic samples measuring 5 cm from the anal rim were taken. After removal, the colon was opened longitudinally and gently washed with 0.9% saline solution. After macroscopic analysis and myeloperoxidase (MPO) and basal transepithelial electrical resistance (TER) assays, samples with the greatest damage were selected to perform the other experiments (Figure 1). The animals were not treated with SC-560 or celecoxib; these COX inhibitors were only used *in vitro*.

### Macroscopic injury scores

Colonic samples were stretched on a flat surface, and scores were assigned according to the level of damage observed using the technique described by Morris et al. (13), which evaluates the presence of hyperemia, ulceration, and thickening of the colonic wall, in addition to the length and number of lesions.

### Wet weight of the colon

Colonic samples were weighed on a precision scale and the results are reported in g/cm of colon (15).

### Microscopic lesion scores

Histological slides were made from colon samples, stained with hematoxylin/eosin, and analyzed by an experienced, blinded pathologist (P.M.G.S.). Histopathological evaluation was performed according to the criteria described by Appleyard and Wallace (16), with scores being assigned according to the observed colonic damage. The parameters analyzed were loss of mucosal architecture (score 0-3), cellular infiltration (score 0-3), muscle thickening (score 0-3), crypt abscess formation (score 0-1), and absence of goblet cells (score 0-1).

### Myeloperoxidase activity

Colonic samples were collected, weighed, macerated in 0.5% hexadecyltrimethylammonium bromide (HTAB) (pH 6.0), and centrifuged (at 1677 *g* for 7 min and 4°C). Next, 10 µL of the supernatant was placed in 96-well plates in duplicate. In each well, 200 µL of a solution containing O-dianisidine (5 mg diluted in 3 mL phosphate buffer) was added. To this solution, 15 μL of 1% H_2_O_2_ was added. Absorbance was determined using an absorbance reader (BMG Labtech, Spain) at a wavelength of 450 nm. The results are reported in units of MPO/mg of tissue (17).

### mRNA expression of colonic tight junctions

The rat colon was used to extract total RNA using the RNeasy Lipid Tissue Mini Kit (Qiagen, Germany) following the manufacturer's instructions. Subsequently, the extracted RNA was reverse transcribed into cDNA using the iScript cDNA Synthesis Kit (Bio-Rad, USA). The expression levels of junction proteins (ZO-1, occludin, claudin-1, and claudin-2) were analyzed using quantitative real-time polymerase chain reaction (qPCR) with the CFX96 Touch Detection System (Bio-Rad). The reference gene YWHAZ, encoding the tyrosine 3-monooxygenase/tryptophan 5-monooxygenase activation protein zeta, was used for normalization. DNA primers specific to the target genes were designed based on mRNA sequences obtained from the National Center for Biotechnology Information (http://www.ncbi.nlm.nih.gov). The CFX Manager 3.0 software (Bio-Rad) was used to determine the relative expression levels based on the threshold cycle (Ct) values, with observed fluorescence levels 10 times higher than the baseline fluorescence for each qPCR assay. The mRNA expression was calculated using the 2^-ΔΔCt^ method (18).

### Experimental protocol in the Üssing chamber

Colonic samples were stretched on a petri dish containing Krebs solution (145 mM NaCl, 0.4 mM KH_2_PO_4_, 1.6 mM K_2_HPO_4_, 5 mM glucose, 1 mM MgCl_2_, and 1.2 mM CaCl_2_; pH 7.4) and dissected using the mucous layer for mounting in the Üssing chamber (Mussler Scientific Instruments, Germany). Sections of the colonic mucosa had an exposure area of 0.017 cm^2^, and the Krebs solution (3.5 mL/semi-chamber) was kept aerated with a carbogenic mixture (95% O_2_ and 5% CO_2_) at a constant temperature of 37°C (12). TER and epithelial permeability to fluorescein were then measured.

### Transepithelial electrical resistance

TER was calculated according to Ohm's law and the methodology described by Tobey et al. (19). After 30 min, the electrical system was stable, and it was possible to identify the basal TER, represented by Ω×cm^2^. The luminal side of the colonic samples was then exposed to the “test solution”, composed of Krebs solution containing ASA (30.8 mM) (12), COX-1 inhibitor (SC-560, 500 nM), or COX-2 inhibitor (Celecoxibe, 500 nM) (20), with each of these substances being diluted in ethanol (etOH), so that etOH had to be used in the Sham group. TER was assessed over the next 60 min. The results are reported as percentage (%) of resistance variation at 0, 15, 30, 45, and 60 min. The treatment was only performed *in vitro.*

### Fluorescein permeability

Paracellular epithelial permeability was analyzed after exposing the tissue to the test solution. Colonic tissues were kept in the Üssing chambers and the solution in the luminal side was replaced with a solution containing fluorescein (1 mg/mL, 376 Da, diluted in Krebs solution with pH 7.4), a permeable fluorescent tracer that passes through mucosal layers (19). Permeability was assessed at 30 min intervals for 90 min from samples (100 μL) collected from the non-luminal side. Fluorescence was quantified using a fluorescence reader (FLUOstar Omega; BMG Labtech, Germany). Fluorescein flux values are reported as a ratio of fluorescein intensity (Tn/T0) from a standard curve constructed for each analysis, where T0 is the initial time and Tn is the analyzed time.

### Statistical analysis

The Shapiro-Wilk test was performed to verify the normality of the samples. For parametric data, Student's *t*-test and analysis of variance (ANOVA) followed by the Bonferroni post-test were used and Kruskal-Wallis test followed by the Dunn's test were used for non-parametric data. Data are reported as means±standard error of the mean (SEM). Statistical significance was set at P<0.05. The number of samples was equal the number of animals, since each animal provided a sample for macroscopic colonic damage, a sample for wet weight, a sample for MPO, a sample for histopathology, and a sample for TER. The software Jamovi 2.3 (https://www.jamovi.org) was used for statistics.

## Results

### Colonic macroscopy damage, wet weight, and MPO concentration

The 7th day colitis group had higher macroscopic damage scores (20.37±1.21) compared to the sham group (0.5±0.22), in addition to a higher wet weight (0.45±0.06 *vs* 0.13±0.01 g/cm of colon, respectively) and MPO activity (73.5±10.2 *vs* 13.6±2.6 UMPO/mg tissue, respectively). However, the 14th and 28th day colitis groups did not show statistical differences in any of these three parameters compared with the sham group (Table 1).

**Table 1:** Colonic macroscopic damage, wet weight, and MPO concentration.

Experimental groups	Macroscopic lesion score	Wet weight(g/cm of colon)	MPO(UMPO/mg of tissue)
Sham (n=7)	0.5±0.2	0.13±0.01	13.6±2.6
Colitis at 7th day (n=8)	20.4±1.2*	0.45±0.06*	73.5±10.2*
Colitis at 14th day (n=8)	1.7±0.9^#^	0.21±0.03^#^	26.5±8.7^#^
Colitis at 28th day (n=8)	2.2±1.1^#^	0.19±0.01^#^	17.6±4.7^#^

Data are reported as means±SEM. *P<0.05 *vs* sham group and ^#^P<0.05 *vs* colitis group at 7th day (one-way ANOVA test followed by Bonferroni test). MPO: myeloperoxidase; U: units.

### Histopathologic damage in TNBS-induced colitis

**Figure 2:**
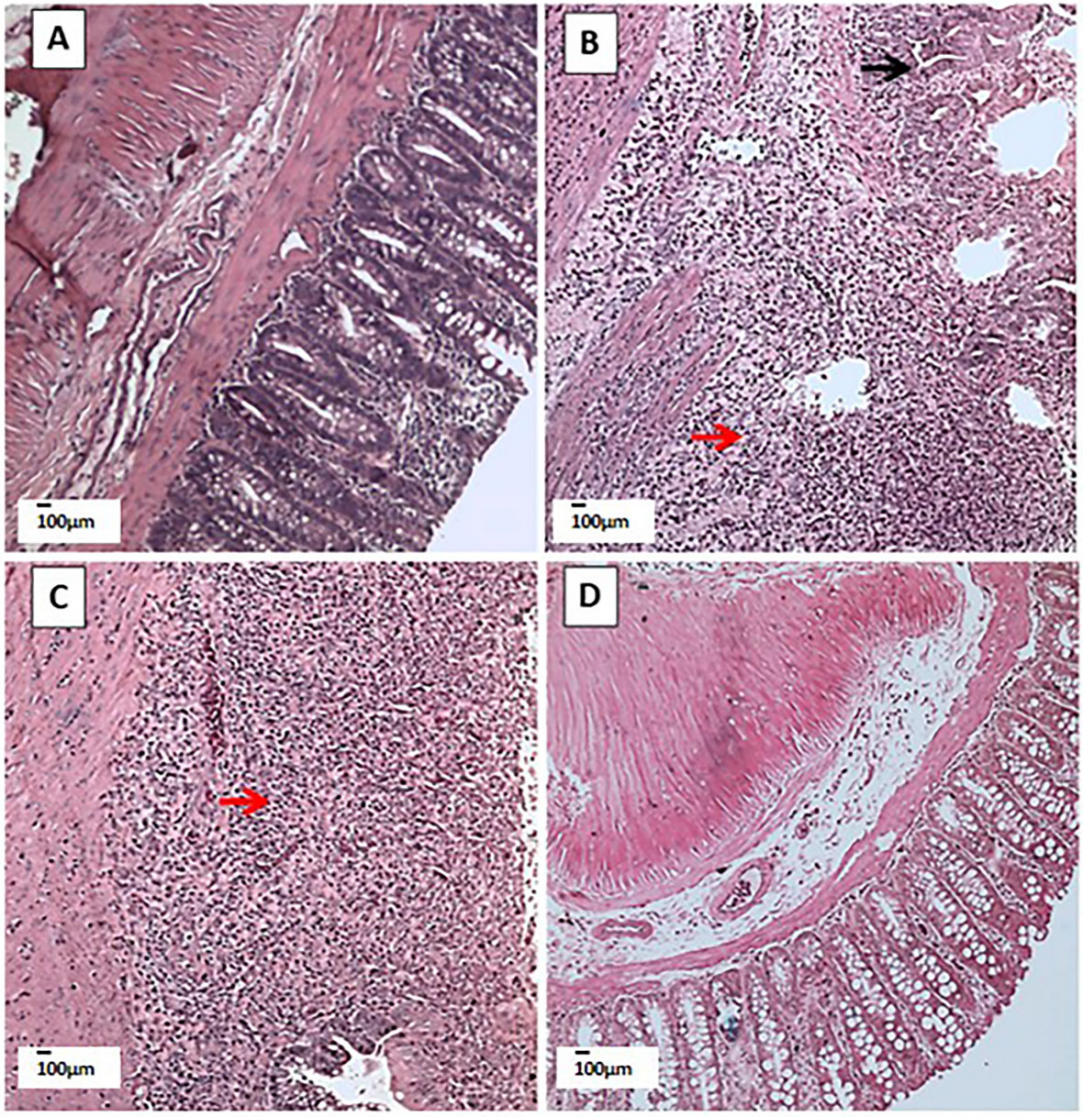
Microscopic damage in TNBS-induced colitis. Representative images of colonic tissues stained with hematoxylin/eosin. Micrographs (scale bar 100 μm) representing the colon of a sham animal (**A**), 7th day colitis animal (**B**), 14th day colitis animal (**C**), and 28th day colitis animal (**D**). Black arrow: loss of mucosal architecture; red arrow: cellular infiltration. TNBS: 2,4,6,-trinitrobenzenesulfonic acid.

Histopathological evaluation showed that the 7th day colitis and 14th day colitis groups presented changes in the microscopic criteria such as loss of mucosal architecture, cellular infiltration, muscle thickening, and absence of goblet cells compared to the Sham group. However, the 28th day colitis group did not present statistical differences in any criterion compared to the Sham group (Table 2) (Figure 2).

**Table 2:** Microscopic colonic damage in TNBS-induced colitis.

Experimental groups	Criteria				
	Loss of mucosal architecture(0-3)	Cellular infiltration(0-3)	Muscle thickening (0-3)	Crypt abscess(0-1)	Goblet cell depletion (0-1)
Sham (n=5)	1 (1-2)	1 (1-2)	1 (1-2)	1 (0-1)	0 (0-0)
Colitis at 7th day (n=8)	3 (1-3)*	3 (2-3)*	3 (2-3)*	1 (1-1)	1 (0-1)*
Colitis at 14th day (n=8)	3 (1-3)*	3 (1-3)*	2 (1-3)	1 (1-1)	1 (0-1)*
Colitis at 28th day (n=8)	1.5 (1-2)	1 (1-2)^#^	1 (0-2)^#^	1 (0-1)	0 (0-0)^#^

Data are reported as median with minimum and maximum in parentheses. *P<0.05 *vs* Sham group. ^#^P<0.05 *vs* colitis group at 7th day (Kruskal-Wallis test followed by Dunn's test).

### Assessment of colonic mucosal integrity using basal TER

**Figure 3:**
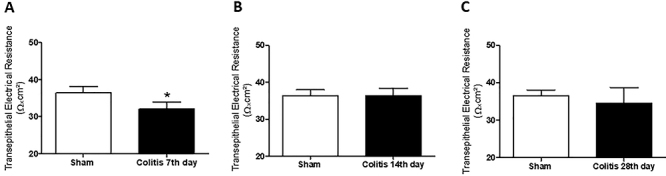
Basal transepithelial electrical resistance (TER) was analyzed in colitis-induced and euthanized animals after 7 days (**A**), 14 days (**B**), and 28 days (**C**). Data are reported as means±SEM (n=20 per group). *P<0.05 *vs* sham group (unpaired Student's *t*-test).

A lower baseline TER was observed in the 7th day colitis group (32.0±1.9 Ω×cm^2^) compared to the Sham group (36.4±1.6 Ω×cm^2^) (Figure 3A). However, the 14th day (36.3±2.1 Ω×cm^2^) and 28th day (34.5±4.2 Ω×cm^2^) colitis groups did not demonstrate statistically different results from the sham group (Figure 3B and C).

### mRNA expression of colonic tight junctions

**Figure 4:**
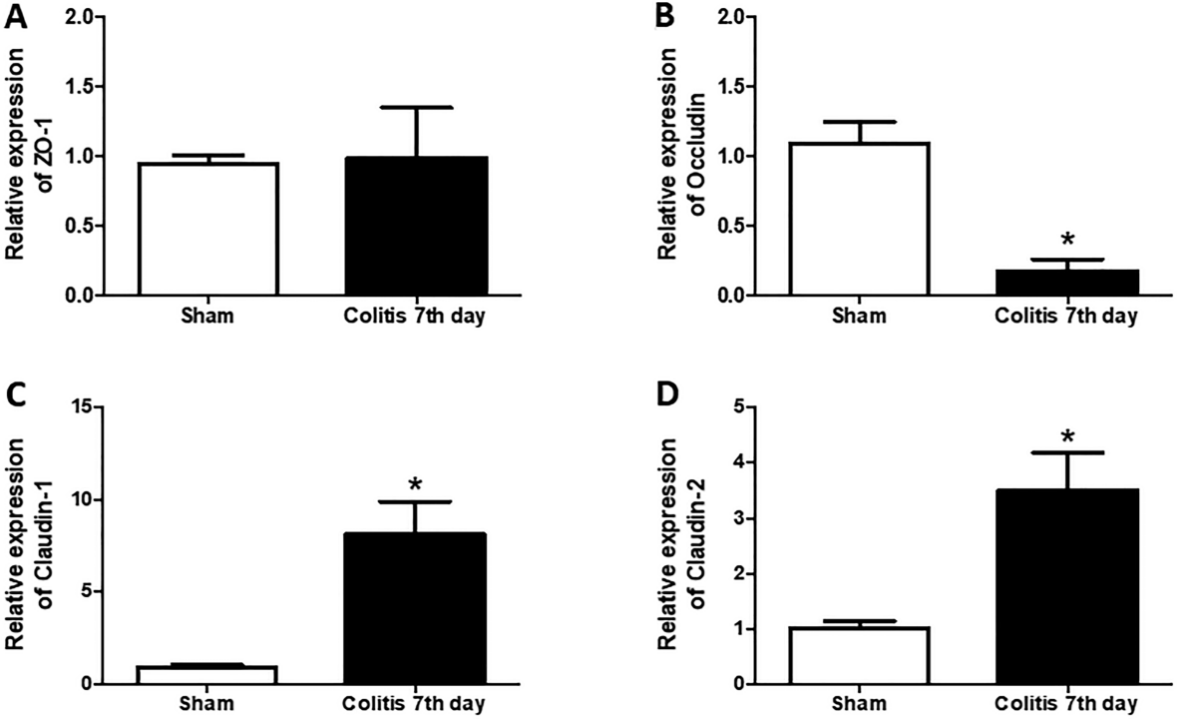
mRNA expression of colonic tight junctions. The mRNA expression of junction proteins was analyzed using quantitative real-time polymerase chain reaction. **A**, zona occludens (ZO)-1; **B**, occludin; **C**, claudin-1; and **D**, claudin-2. Data are reported as means±SEM (n=8 per group). *P<0.05 *vs* Sham group (unpaired Student's *t*-test).

The colitis group showed lower expression of the junction protein occludin compared to the Sham group (0.18±0.08 *vs* 1.09±0.16, respectively, P<0.05) (Figure 4B). The colitis group had higher gene expression of claudin-1 and claudin-2 compared to the Sham group (8.12±1.79 *vs* 0.94±0.16 and 3.51±0.67 *vs* 1.01±0.13, respectively, P<0.05) (Figure 4C and D). No statistical difference in the relative expression of zona occludens-1 (ZO-1) was observed between the groups (Figure 4A).

### Acetylsalicylic acid decreased colonic mucosal integrity

**Figure 5:**
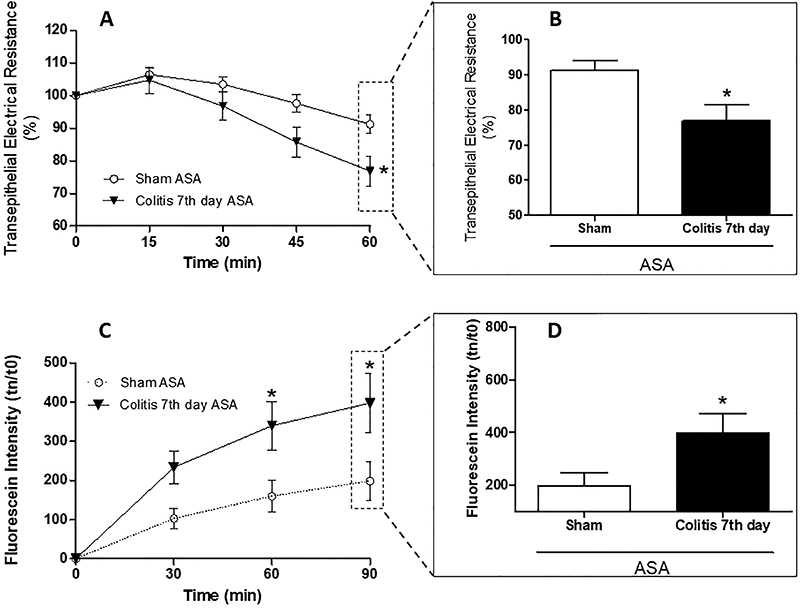
Acetylsalicylic acid (ASA) decreases colonic mucosal integrity. Transepithelial electrical resistance (TER) was assessed after mucosal exposure to ASA at 0, 15, 30, 45, and 60 min (**A**). TER at 60 min (**B**). After 60 min of ASA exposure, fluorescein samples were collected at 0, 30, 60, and 90 min (**C**). Permeability of fluorescein at 90 min (**D**). Data are reported as means±SEM (n=10 per group). *P<0.05 *vs* sham group [two-way analysis of variance test followed by Bonferroni post-test (**A** and **C**) and unpaired Student's *t*-test (**B** and **D**)].

The 7th day colitis group showed a decrease in TER compared to the Sham group after both groups were exposed to ASA (Figure 5A), showing a greater drop after 60 min of exposure (76.85±4.66 *vs* 91.21±2.79%, respectively) (Figure 5B). In agreement with this result, there was an increase in colonic mucosa permeability in the 7th day colitis group after ASA challenge compared to the Sham group (Figure 5C), with greater passage of fluorescein at 90 min (339.7±61.88 *vs* 198.4±49.43 fluorescein intensity, respectively) (Figure 5D).

### SC-560, but not celecoxib, decreased colonic mucosal integrity

**Figure 6:**
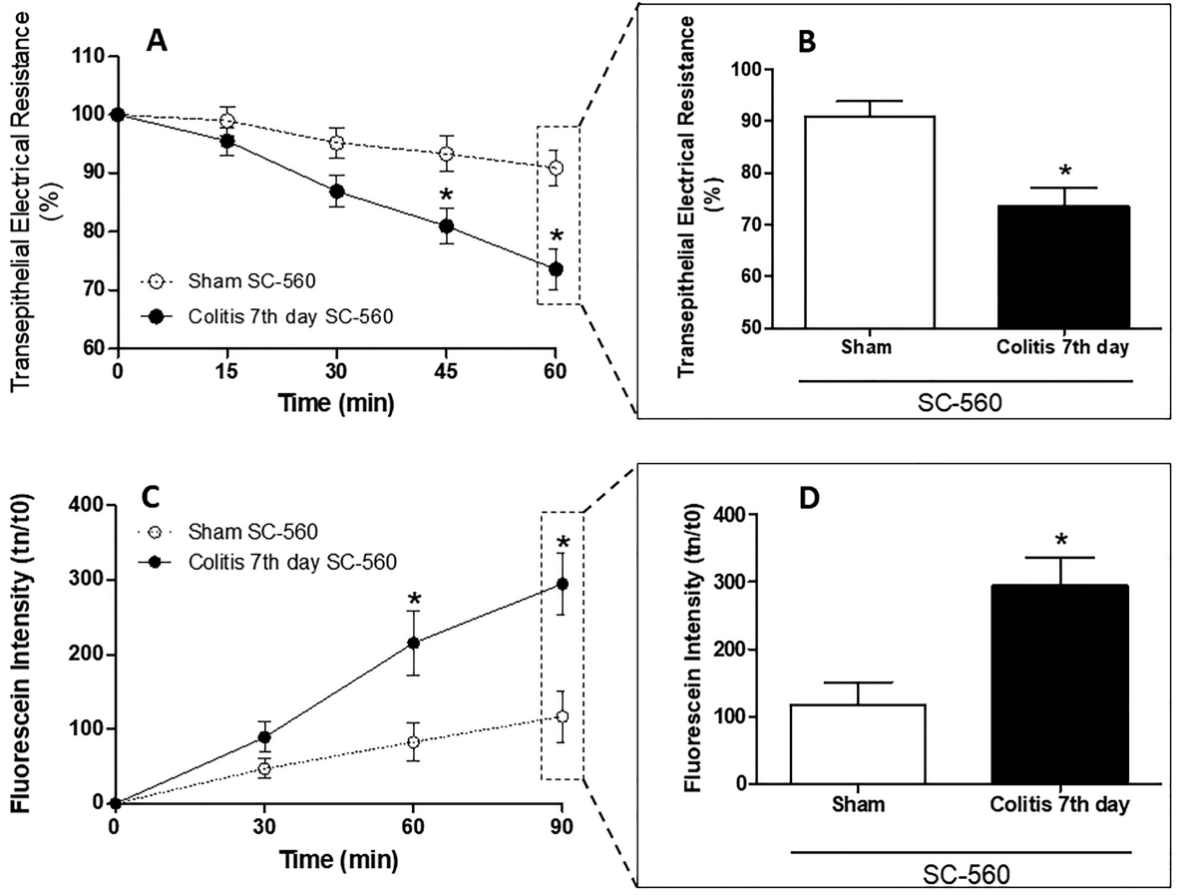
SC-560 decreases colonic mucosal integrity. Transepithelial electrical resistance (TER) was evaluated after mucosal exposure to a selective COX-1 inhibitor at 0, 15, 30, 45, and 60 min (**A**). TER drop at 60 min (**B**). After exposure to SC-560 for 60 min, fluorescein samples were collected at 0, 30, 60, and 90 min (**C**). Permeability of fluorescein at 90 min (**D**). Data are reported as means±SEM (n=8 per group). *P<0.05, two-way analysis of variance test followed by Bonferroni post-test (**A** and **C**) and unpaired Student's *t*-test (**B** and **D**).

**Figure 7:**
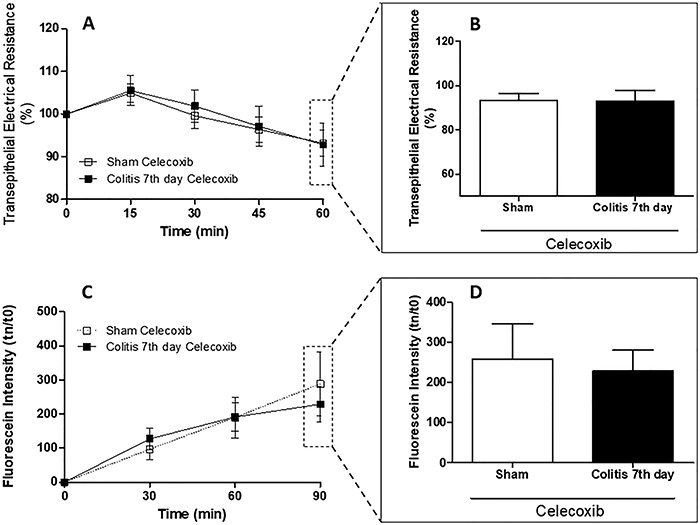
Celecoxib does not affect colonic mucosal integrity. Transepithelial electrical resistance (TER) was evaluated after mucosal exposure to a selective COX-1 inhibitor at 0, 15, 30, 45, and 60 min (**A**). TER drop at 60 min (**B**). After 60 min of exposure to celecoxib, fluorescein samples were collected at 0, 30, 60, and 90 min (**C**). Permeability of fluorescein at 90 min (**D**). Data are reported as means±SEM (n=8 per group). P>0.05, two-way analysis of variance test followed by Bonferroni post-test (**A** and **C**) and unpaired Student's *t*-test (**B** and **D**).

SC-560 altered the integrity of the colonic mucosa of animals with colitis compared with sham animals. A decrease in TER was observed (Figure 6A), with a maximum after 60 min (73.6±3.5 and 90.9±3.02%) (Figure 6B). In addition, an increase in fluorescein permeability (Figure 6C) was observed, with a maximum after 90 min (294.8±41.97 fluorescein intensity) (Figure 6D). When using celecoxib, in turn, it was not possible to observe changes in the integrity of the colonic mucosa of animals with and without colitis, in both TER (Figure 7A and B) and fluorescein permeability (Figure 7C and D).

## Discussion

IBDs are important because they have a high prevalence and increasing incidence (1). In addition to intestinal manifestations, it is also common to observe extra-intestinal manifestations, especially in joints, which often require the use of NSAIDs for treatment (2). Loss of colonic barrier integrity is important in IBD, with prostaglandins being relevant factors in this process (9). Our data showed that damage to the colonic mucosa in the experimental colitis model by TNBS in rats can be aggravated by the inhibition of COX-1 but not COX-2, thereby demonstrating the importance of this enzyme for homeostasis maintenance. In addition, COX-2 injection did not worsen IBD activity.

Rats had a peak of inflammation on the 7th day after colitis induction and an almost complete reduction of inflammatory parameters 28 days after induction. Thus, as the 7th post-induction day was the most representative of the disease, according to the criteria of inflammation and loss of mucosal integrity, we used this day for subsequent experiments.

In the present study, animals with seven days of colitis induction had greater macroscopic and microscopic damage, neutrophilic infiltration, and loss of mucosal architecture and higher MPO levels than the control group. Other studies found similar results, demonstrating the potent action of TNBS associated with etOH, reproducibility of the experiment, and installation of the inflammatory process on the 7th day after colitis induction (21,14,15). Similar findings were not observed on the 14th or 28th day after induction, possibly due to spontaneous recovery of the inflammatory process.

Although the dysfunction of the intestinal barrier in colitis is not fully understood, the increased inflammatory cytokines in the colonic mucosa are believed to play a critical role in the dysregulation of TJ. In our study, we identified alterations in the expression of some TJ proteins, including a reduction in occludin and an increase in claudins-1 and -2. Studies show that in intestinal mucosa inflammation, there is a reduction of TJs, including occludin and barrier-forming claudins, while there is an increase in pore-forming claudins (22). The elevated expression of claudin-1 may be an attempt of the organism to strengthen the integrity of the intestinal barrier and reduce the passage of unwanted substances through the epithelium, acting as a compensatory mechanism to help maintain the integrity of the TJ complex despite the loss of other junction proteins, such as occludin (23). While our research did not show alterations in ZO-1 levels, another group obtained similar results to ours but demonstrated through immunofluorescence that there was an increase in intercellular gaps and a change in the localization of ZO-1 in inflammatory conditions (24).

Previous studies have shown that COX balance is of fundamental importance in the establishment of physiological conditions, serving as an important defense mechanism for the organism (7). However, our intention was to evaluate the impact of its isoforms on experimental colitis. Thus, we observed that the use of a nonspecific COX inhibitor (ASA) caused a decrease in colonic integrity in animals with colitis, resulting in a decrease in TER and an increase in paracellular permeability. These results are in agreement with previous findings that ASA reduces the integrity of the colonic mucosa, with a decrease in TER and an increase in paracellular permeability in biopsies from patients with CD compared with biopsies from individuals without CD, which is associated with a decrease in the levels of claudin-1, claudin-5, and tricellulin components of the TJs (12).

Suspecting that the changes caused by ASA were due to inhibition of COXs, we investigated whether an imbalance of these enzymes could cause such changes. The COX-1 isoform is constitutively expressed in most tissues and is essential for the maintenance of its physiological state, including the protection of the gastrointestinal mucosa (25-27). Initially, our group observed that SC-560 reduced the colonic integrity of animals with colitis, resulting in a decrease in TER and an increase in fluorescein permeability compared to the sham group exposed to the same inhibitor. These results can be explained by the blocking of COX-1 in the gastrointestinal mucosa, which leads to inhibition of the production of prostacyclin and prostaglandins PGE_2_ and PGD_2_ (28), which act as cytoprotective agents in the gastrointestinal mucosa (29), and their inhibition may decrease the integrity of the mucosa. However, we did not evaluate whether inhibition of COX-1 is associated with repair and proliferation/apoptosis because it would have been necessary to treat the animals with a COX-1 inhibitor during the course of inflammation, which was not performed.

COX-2, but not COX-1, is essential for inflammation, and this is due to several stimuli, including cytokines, endotoxins, and growth factors, and is also responsible for inducing prostaglandins that contribute to the development of inflammation signs (30-32), which contribute to the loss of tissue integrity (5). In the present study, we observed that there was no change in the integrity of the colonic mucosa after exposure to celecoxib. Thus, our results agreed with data described in the literature on the inflammation-inducing effect of this enzyme, and its inhibition helps maintain the integrity of the colonic mucosa. Thus, patients who need prolonged use of anti-inflammatory drugs can use selective coxibs because of their greater safety. Other studies should be performed to confirm our hypothesis.

Previous papers have reported that COX-1 is responsible for prostaglandin production in the early inflammatory process, while COX-2 becomes the main driver for prostaglandin synthesis as inflammation progresses (33,34). We agree that study timing is important for defining the role of COX during inflammation. However, in our experiment, inflammation started 7 days earlier, so at this time both COX-1 and COX-2 are expressed.

We acknowledge that our study had certain limitations. First, we did not administer SC-560 or celecoxib to the animals. However, this decision was made to avoid masking the exacerbation of colitis and hindering functional assessment. Second, we did not determine the expression of COX enzymes in the colon. Nevertheless, previous studies have demonstrated an upregulation of COX-2 expression during inflammation (35-37). It is important to note that *in vitro* testing does not fully represent the response of a living organism to a drug, nor does it account for potential responses from other organs and systems beyond the specific tissue under study (38). For instance, when assessing *in vivo* intestinal permeability using fluorescein isothiocyanate-dextran, the marker's passage is influenced by the actions of and interactions with other organs within the whole organism (39). However, in our specific model, it was not feasible to use certain methods to modulate the metabolic pathway, such as the selective COX-1 inhibitor employed in our work, as it could potentially harm the human body, given that COX-1 is constitutively expressed. Therefore, the *ex-vivo* study carried out holds significant importance in this context.

This study suggested that damage to the integrity of the colonic mucosa in the experimental colitis model induced by TNBS in rats can be aggravated by inhibiting COX-1, which demonstrated the importance of this enzyme in the maintenance of homeostasis characterized by TER and permeability of the intestinal mucosa.
